# Full-Length Genome Analyses of Two New Simian Immunodeficiency Virus (SIV) Strains from Mustached Monkeys (*C. Cephus*) in Gabon Illustrate a Complex Evolutionary History among the SIVmus/mon/gsn Lineage

**DOI:** 10.3390/v6072880

**Published:** 2014-07-22

**Authors:** Florian Liégeois, Fabian Schmidt, Vanina Boué, Christelle Butel, Fatima Mouacha, Paul Ngari, Bertrand Mve Ondo, Eric Leroy, Jonathan L. Heeney, Eric Delaporte, Martine Peeters, François Rouet

**Affiliations:** 1 UMI 233, Institut de Recherche pour le Développement (IRD) and University of Montpellier I, Montpellier 34396, France; E-Mails: svetnina@yahoo.fr (V.B.); christelle.butel@ird.fr (C.B.); fatima.mouacha@ird.fr (F.M.); eric.delaporte@ird.fr (E.D.); martine.peeters@ird.fr (M.P.); 2 Centre International de Recherches Médicales de Franceville, Franceville BP 769, Gabon; E-Mails: paul_ngari@yahoo.fr (P.N.); mvebertrand2000@yahoo.fr (B.M.O.); eric.leroy@ird.fr (E.L.); frouet@pasteur-kh.org (F.R.); 3 Department of Veterinary Medicine, University of Cambridge, Cambridge CB3 0ES, UK; E-Mails: fs335@cam.ac.uk (F.S.); jlh66@cam.ac.uk (J.L.H.)

**Keywords:** SIV, lentivirus, non-human primate, molecular phylogeny, evolution, bushmeat, cross-species transmission, Gabon

## Abstract

The Simian Immunodeficiency Virus (SIV) mus/mon/gsn lineage is a descendant of one of the precursor viruses to the HIV-1/SIVcpz/gor viral lineage. SIVmus and SIVgsn were sequenced from mustached and greater spot nosed monkeys in Cameroon and SIVmon from mona monkeys in Cameroon and Nigeria. In order to further document the genetic diversity of SIVmus, we analyzed two full-length genomes of new strains identified in Gabon. The whole genomes obtained showed the expected reading frames for *gag*, *pol*, *vif*, *vpr*, *tat*, *rev*, *env*, *nef*, and also for a *vpu* gene. Analyses showed that the Gabonese SIVmus strains were closely related and formed a monophyletic clade within the SIVmus/mon/gsn lineage. Nonetheless, within this lineage, the position of both new SIVmus differed according to the gene analyzed. In *pol* and *nef gene*, phylogenetic topologies suggested different evolutions for each of the two new SIVmus strains whereas in the other nucleic fragments studied, their positions fluctuated between SIVmon, SIVmus-1, and SIVgsn. In addition, in C1 domain of *env*, we identified an insertion of seven amino acids characteristic for the SIVmus/mon/gsn and HIV‑1/SIVcpz/SIVgor lineages. Our results show a high genetic diversity of SIVmus in mustached monkeys and suggest cross-species transmission events and recombination within SIVmus/mon/gsn lineage. Additionally, in Central Africa, hunters continue to be exposed to these simian viruses, and this represents a potential threat to humans.

## 1. Introduction

Human Immunodeficiency Viruses Type 1 and 2 (HIV-1 & -2) stem from Simian Immunodeficiency Viruses (SIV) infecting apes from West Central Africa and sooty mangabeys from West Africa, respectively [1]. Beyond these well-described SIVs, a plethora of non-human primates (NHPs) in sub-Saharan Africa are SIV carriers [2]. Each NHP species is generally infected with a species-specific SIV, *i.e*., multiple strains from the same host species form a monophyletic clade. In some cases, closely related monkey species harbor also closely related SIVs, suggesting that some of these viruses may have coevolved with their hosts for an extended period of time or that SIVs could be transmitted preferentially according to a host-switching model, e.g., l'hoest and sun-tailed monkeys from the l’hoesti superspecies, the four species of African green monkeys (genus *Chlorocebus*) or SIVs from arboreal *Cercopithecus* species [3,4,5]. A single NHP species can also be infected by two different SIVs, e.g., SIVmnd-1 and -2 in mandrills, which are geographically separated by the Ogooué River, but co-circulating SIV variants have also been observed, e.g., SIVmus-1 and -2 in Cameroonian mustached monkeys (*C. cephus cephus*) [6,7]. Recently, a divergent SIVmus strain was partially identified in mustached monkeys (*C. cephus cephus*) inhabiting Gabon [8]. Thus, mustached monkeys were the first monkey species known to date to carry three different SIV lineages [8].

There are also many examples of cross-species transmissions of SIVs between NHPs sharing the same habitat, for example SIVagm infecting African green monkeys has been transmitted to Patas monkeys in West Africa and to yellow and chacma baboons in Eastern and Southern Africa [9,10,11]. Cross-species transmission followed by recombination between different SIV strains is not unusual, as demonstrated for SIVmus-2 infecting mustached monkeys from Cameroon, a virus resulting from the recombination of SIVgsn infecting greater spot-nosed monkeys and SIVmus infecting mustached monkeys [7]. SIVcpz infecting chimpanzees is another example; SIVcpz is a recombinant or “chimeric” virus, with viruses from at least two monkey species contributing. Phylogenetic analyses comparing topologies in the different genes of the genome revealed that most of the 3’ half of SIVcpz (*vif* to *env*) is closely related to the SIVmus/mon/gsn lineage identified in mustached (*C. cephus*), mona (*C. mona*) and greater spot-nosed guenons (*C. nictitans*), respectively [12,13]. The 5’ half of SIVcpz (*gag* and *pol*) is most closely related to SIVrcm identified in red-capped mangabeys (*Cercocebus torquatus*) [13,14]. Further investigation of the phylogenetic topology showed that the *nef* gene is also derived from red-capped mangabeys [15]. The resulting chimeric virus has lost its overlapping reading frame between *env* and *nef* [13]. Overall, these observations indicate that both cross-species transmission and co-infections, followed or not by recombination events, with highly divergent lentiviral strains are possible and that the evolutionary history of NHP lentiviruses has been driven by these successive events over an extended period of time.

Uniquely among the NHP lentiviruses, a subset of guenon (*Cercopithecus*) and all ape SIVs carry the additional accessory gene *vpu* [12]. While absent in the vast majority of characterized guenon SIVs, the homology of *vpu* with a subdomain of the primate TASK-1 channel led to the speculation that it was acquired by molecular piracy during the evolution of SIV in one of the *Cercopithecus* species [16].

Here we present full-length genomes derived from SIVmus strains identified outside of Cameroon. Both highly divergent isolates were identified in Gabon, one in a central region close to the Ogooué River [8] and the other in the east, in close proximity to the border with the Republic of Congo. Furthermore, while describing their phylogenetic relationship to other SIVs, we focus on the relationships with SIVs infecting mustached, greater spot nosed and mona monkeys and observed a domain in *env,* that has so far been neglected.

## 2. Materials and Methods

### 2.1. Non-Human Primate Specimens, Serologic testing, DNA Extractions and SIV PCR Screening

**Figure 1:**
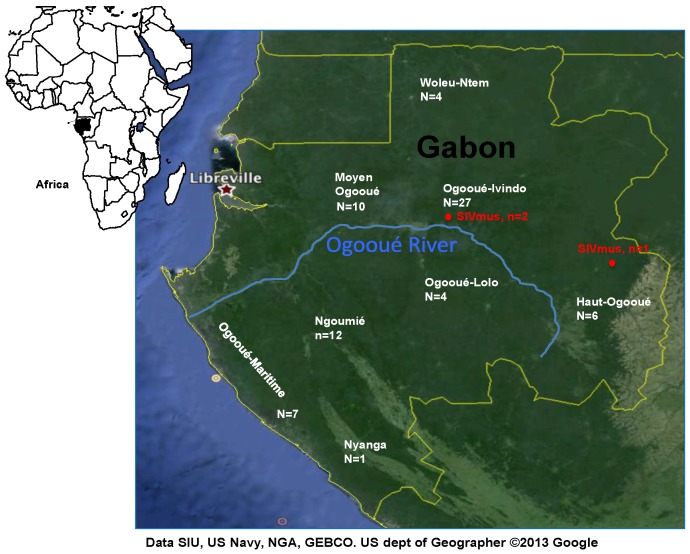
Geographical distribution of samples from moustached monkeys in Gabon. **Haut-Ogooué** (HO) *n* = 6, **Moyen Ogooué** (MO) *n* = 10, **Ngounié** (NG) *n* = 12, **Nyanga** (NY) *n* = 1, **Ogooué Ivindo** (OI) *n* = 27, **Ogooué Lolo** (OL) *n* = 4, **Ogooué Maritime** (OM) *n* = 7, **Woleu-Ntem** (WN) *n* = 4. Red points show the two sites where SIVmus positive animals were identified.

Between 2009 and 2011, whole blood (*n* = 25) and/or tissue (*n* = 46) samples were obtained from 71 wild-caught mustached monkey carcasses in different locations situated in both parts of the Ogooué River in Gabon, which is a geographic barrier for the SIVmnd-1 and mnd-2 viruses in mandrills (Figure 1). Necropsies were performed on sampling sites. All animals were sampled between 2 and 24 h after death. To avoid autolysis of the collected tissues, thin sections were either snap frozen in liquid nitrogen before they were stored at −80 °C in the laboratory. Four out of the 71 samples were collected from juvenile animals (<2 years). Species were initially determined by visual inspection according to the Kingdon Field Guide to African Mammals [17] and the taxonomy described by Colin Groves [18]. All NHP samples were obtained with the authorization of provincial inspections of Water and Forests and the Centre National de la Recherche Scientifique et Technologique (CENAREST, AR0031/09, AR0006/11).

For all samples, total DNA was extracted from whole blood and/or tissues with the QiAamp^®^ blood and QiAamp^®^ tissue kit, respectively, according to the manufacturer’s instructions (Qiagen S.A, Courtaboeuf, France). Sample DNA qualities were checked and primate species were confirmed by amplification of mitochondrial (mt) 12sRNA gene as previously described [19]. Using Basic Local Alignment Search Tool [20] and phylogenetic analysis, mitochondrial sequences obtained were then compared to available genomic data.

All 25 whole blood samples were tested for the presence of SIV antibodies with an in-house SIVmus/mon/gsn lineage-specific ELISA based assay using a synthetic V3-loop peptide as antibody capture [21].

All SIV antibody positive samples and all tissue samples were tested for the presence of SIV infection by amplification of a small *pol* fragment (300 bp) with a set of degenerate consensus primers as previously described (Table S1: Additional File) [22]. PCR positive samples were then sequenced and compared to representatives of the other known SIV lineages by phylogenetic analysis.

### 2.2. PCR Amplification and Sequencing of New SIVmus Full-Length Genomes

Similarly as for previous reports on full-length characterization of new SIVs [23,24,25], new SIVmus full-length genomes were obtained by amplification of overlapping PCR fragments and unintegrated circular DNA using combinations of new SIVmus and SIVmus/mon/gsn lineage specific primers as well as SIV generic primers. The primers used are shown in Table S1.

PCR amplifications were performed using the Long Expand PCR kit (Roche Applied Science, Indianapolis, IN, USA) according to the manufacturer’s instructions. Each amplification reaction included a manual hot-start followed by 35 to 40 cycles. Annealing temperatures were set according to the primer melting temperatures, and extension times varied depending on the size of the expected fragment and were typically set at 1mn/kb. PCR products were agarose gel purified and directly sequenced in both 5' and 3' directions using cycle sequencing and dye terminator methodologies (ABI PRISM Big Dye Terminator Cycle Sequencing Ready Reaction kit with Amplitaq FS DNA polymerase (PE Biosystems, Warrington, England, UK) on an automated capillary sequencer (ABI 3130*XL*, Applied Biosystems, Foster City, CA, USA). To reconstitute the full-length genome sequence, overlapping sequences were assembled into contiguous sequences using SEQMAN DNASTAR software (lasergene, DNASTAR, Inc., Madison, WI, USA).

### 2.3. Sequence Similarity Plots, Bootscan Analyses, and Genetic Identities

Partial and full-length non-concatenated nucleic acid sequences of the new SIVs were aligned using MEGA 5 [26], with minor manual adjustments. Sites that could not be unambiguously aligned were excluded and divergent regions were excluded from subsequent analyses. In order to study whether the newly characterized SIVmus sequences were recombinant with any of the other SIV/HIV lineages, similarity plot analysis was performed with the SIMPLOT package version 2.5 [27] using a sliding window of 500 nucleic acids (na) moved in steps of 50 na. Bootscan analyses were also performed using a nucleic acid alignment of SIVs infecting only arboreal *Cercopithecus* monkeys with the SIMPLOT package version 2.5 [27] using a sliding window of 500 nucleic acids (na) moved in steps of 50 na. Genetic identity analyses were realized using ClustalX [28].

### 2.4. Phylogenetic Analyses

Phylogenies were inferred using both Bayesian methods (implemented in Mr Bayes v3.1) [29] and Maximum Likelihood (ML) method (implemented in PhyML) [30]. Mr Bayes ran for four, three, and three million generations for *gag*, *pol*, and *env* genes, respectively, with a 10% burn-in. In ML method, the reliability of branching orders was tested using the bootstrap approach (1000 replicates). Bayesian parameters were examined with the Tracer program [31]. The suited evolution model (GTR + Γ_4_ + I) was defined using Topali [32]. Substitution saturation’s index tests were performed using Xia’s method implemented in DAMBE software V.5.3.109 [33].

### 2.5. RNA Secondary Structure and Protein Surface Exposure Predictions

The TAR RNA secondary structure was predicted and drawn using the GENQUEST DNASTAR package (Lasergene, DNASTAR Inc., Madison, WI, USA). Protein surface exposure was predicted using NetSurfP v1.1 [34].

### 2.6. Nucleotide Sequence Accession Numbers

The complete sequences have been deposited to the GenBank under the following numbers: KF304707 for SIVmus-09Gab-OI81, KF304708 for SIVmus-11Gab-Pts02.

## 3. Results

### 3.1. SIV Antibodies and PCR Screening in Mustached Monkeys

Whole blood (*n* = 25) and tissue (*n* = 46) samples were obtained from 71 wild-caught mustached monkey carcasses in different locations in Gabon (Figure 1). Out of the 25 whole blood samples that were accessible for serological testing, a single sample (OI81) showed cross-reactivity with a specific SIVmus/mon/gsn peptide derived from the V3-loop of the envelope gene (Optical density > 1.4, data not shown). For this sample and two (Pts02, OIF02) of 46 additional DNA samples extracted from lymph nodes, an SIV *pol* PCR fragment (300 bp) was successfully amplified and compared to representative SIV lineages known to date (Figure S1). All new SIVmus strains from Gabon formed a specific clade (Figure S1) within the SIVmus/mon/gsn lineage.

Thus, out of the 71 NHP samples tested, three were confirmed SIV infected leading to a prevalence rate of 4.2%. All SIV positive monkeys originated from the north of the Ogooué River. Two were (OI81, OIF02) from the same site situated in the Ogooue-Ivindo province in the center of Gabon whereas one (Pts02) was from the Haut-Ogooué province in the eastern part of Gabon. Both collecting sites are 250 km apart (Figure 1).

### 3.2. Molecular Species Confirmation

Sequencing of amplicons in the mitochondrial 12sRNA confirmed that all 71 samples were derived from *C. cephus* monkeys. Nonetheless, phylogenetic analyses allowed us to distinguish between two *C. cephus* subspecies that are naturally found in Gabon (Figure S2). *C. cephus cephus* is found north of the Ogooué River whereas *C. cephus cephodes* is present in the south: three samples (OM28, OM29, OM31) collected south of the Ogooué River and initially considered as *C. c. cephodes* subspecies were finally classified as being from the *C. c. cephus* subspecies. Likewise, two samples (MO271, MO11) collected north of the Ogooué River were likely hunted on the opposite riverbank. Indeed, villagers living along the river often cross the Ogooué to hunt. Finally, our sampling was composed of 48 *C. c. cephus* and 23 *C. c. cephodes*. The SIV positive samples were from the *C. c. cephus* subspecies (Figure S2).

### 3.3. Genomic Organization and Functional Motifs of New SIVmus Strains

Out of the three new SIVmus strains that were partially amplified, we obtained the full-length genomes for two. We failed to amplify supplementary SIV DNA fragments from the OIF02 sample. It could be due to poor DNA quality, mismatches with the primers used or to the low presence of SIV proviral DNA integrated in cells.

The new SIVmus-09Gab-OI81 (9205 bp) and SIVmus-11Gab-Pts02 (9448 bp) full-length genomes were then compared to other primate lentiviruses and showed the expected reading frames for *gag*, *pol*, *vif*, *vpr*, *tat*, *rev*, *env*, and *nef* and encoded also for a *vpu* gene. The long terminal repeats (LTR) contained all the characteristic features of other primate lentivirus LTRs, including TATA, NF-kB sites, and potential SP-1 regions. The secondary structure prediction of the TAR element showed an identical organization to the previously described TAR element found in SIVs infecting arboreal *Cercopithecus* species confirming that TAR element organization represents a specific signature of these SIVs (Figure S3) [5].

**Figure 2:**
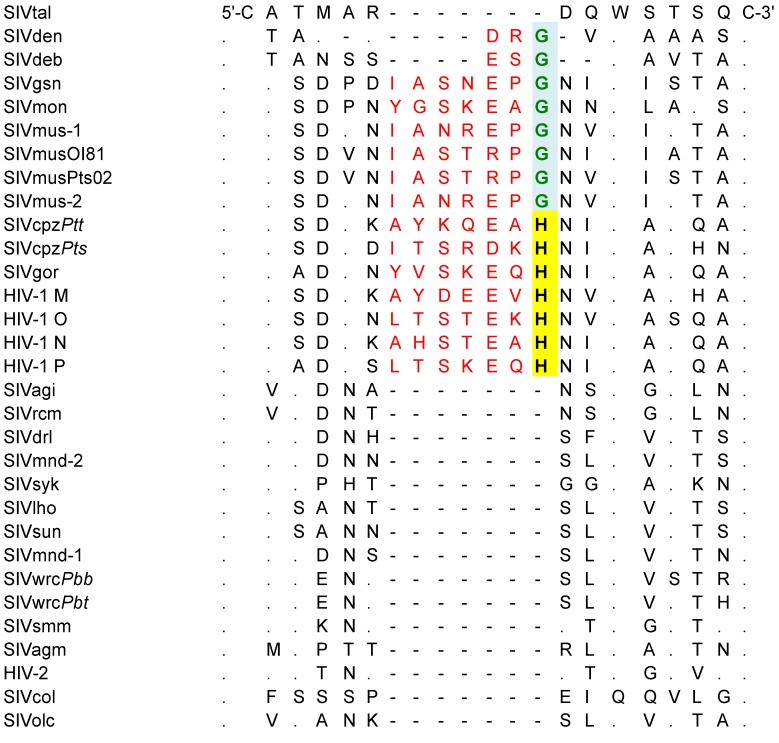
Comparison of C1 domain, between the first and second conserved cysteine residues for different Simian Immunodeficiency Virus (SIV) lineages, Human Immunodeficiency Virus (HIV)-1 and HIV-2. The insertion of seven amino acids present in the SIVmus/mon/gsn lineage, SIVcpz, SIVgor and HIV-1 M, N, O, P is shown in red letters. In SIVs from guenons (*Cercopithecus* species), this domains ends in a glycine (highlighted in green box), while in all hominoid lineages the last amino acid residue of this domain is a highly conserved histidine (highlighted in yellow box). Dots indicate amino acid identity and dashes indicate gaps introduced to optimize the alignment.

Like all other known primate lentiviruses, the new SIVmus strains contain 18 cysteine residues conserved across the gp120 envelope glycoprotein surface subunit. Interestingly, in the C1 domain, between the first and second conserved cysteine residues, we observed an insertion of seven amino acids also present in other SIVs from the SIVmus/mon/gsn and HIV-1/SIVcpz/SIVgor lineages (Figure 2). For SIVmus/mon/gsn, this domain ends with glycine (highlighted in green, Figure 2), while for HIV-1/SIVcpz/SIVgor the last amino acid residue of this domain is a highly conserved histidine (highlighted in yellow box, Figure 2). Unlike the highly conserved domains framing this amino acid septet, this short hydrophilic domain is predicted to be exposed on the gp120 surface. The fifth amino acid of this domain is usually a negatively charged amino acid (Aspartate or Glutamate); however, in both newly described SIVmus strains, we found the polar amino acid serine with an uncharged side chain. Of note, while SIVdeb and SIVden lack these additional seven amino acids, both SIVs carry the highly preserved amino acid triplet, E/D-X-G (Figure 2).

Finally, two different binding sites, known to be critical for primate lentivirus budding have been identified in SIV Gag p6 protein sequences: PT/SAP and YPXL [5]. With the exception of SIVdeb and SIVden, both motifs (PT/SAP and YPXL) are found in *arboreal Cercopithecus*, *Miopithecus*, *Piliocolobus and Procolobus* SIV lineages [5,23,24,35]. As expected, both motifs were present in the new SIVmus strains.

### 3.4. Phylogenetic Analyses of the New SIVmus Strains

**Figure 3:**
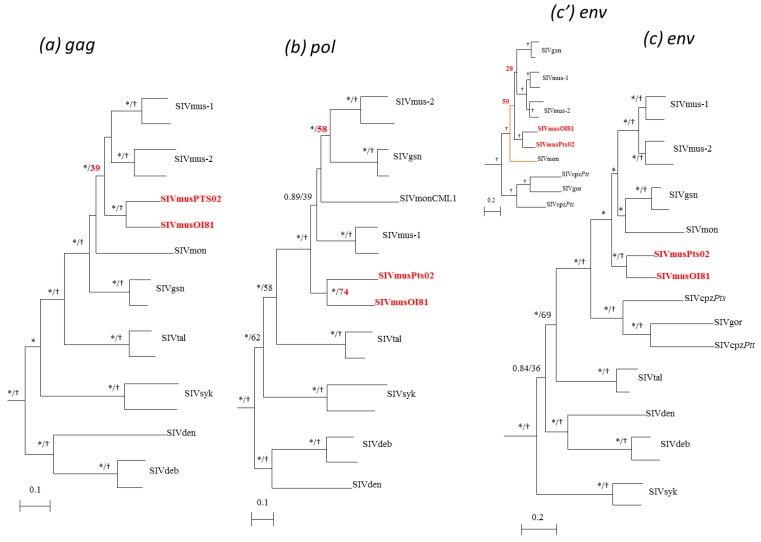
Phylogenetic relationships of the newly derived SIVmus sequences to other SIV lineages in the three major genes. *gag* (*1228 nt*) (***a***), *pol* (*2772 nt*) (***b***), and *env* (*1953*) (***c***). Only sequences of interest are shown. Phylogenies were inferred using both Bayesian and Maximum Likelihood methods implemented in Mr Bayes and PhyML, respectively, under the GTR + Γ_4_ + I model of evolution. Stars and crosses at nodes represent posterior probability (≥90%) and bootstrap values (≥80%), respectively. Scale bars indicate substitution per site. In *env*, the tree topologies differed according the phylogenetic method used. In the figure, (**c**) represents the topology done for *env* by using the bayesian method whereas (**c’**) represents the topology done by using PhyML method.

In order to compare the new full-length SIVmus sequences to previously characterized SIV strains, we first performed phylogenetic analyses using the nucleic acid sequence from the three structural genes: *gag* (1228 na), *pol* (2772 na), and *env* (1953 na). The different *gag*, *pol*, and *env* phylogenetic trees showed that the two new SIVmus strains were closely related to each other and formed a monophyletic clade throughout their entire genomes. These strains represented a new SIVmus lineage that falls within the SIVmus/mon/gsn lineage (Figure 3). Nonetheless, depending on the phylogenetic method used, we observed differences in the reliability of the trees. In *gag* and *env*, Bayesian and ML methods provided high posterior probabilities or bootstrap values, respectively (Figure 3a,c). In the *pol* gene, the Bayesian posterior value was equal to one whereas the bootstrap value was 74% (55% in Pol proteome, data not shown) (Figure 3b). Indeed, ML analysis in the *pol* gene suggested an unclear relationship between the two new SIVmus strains. Furthermore, in the three genes studied, the phylogenetic positions of the new SIVmus strains varied. In the *gag* gene, they clustered at the root of SIVmus-1 and -2, although here too we observed an important difference between posterior probability (>0.9) and bootstrap (<40) values. In *pol* gene, the new SIVmus strains from Gabon were at the root of the SIVmus/mon/gsn lineage and in the *env* gene their positions depended of the phylogenetic method used. Using the Bayesian method, they clustered at the root of SIVmus/mon/gsn lineage (Figure 3c), whereas they were at the root of SIVmus-1 & -2 and SIVgsn with the ML method (Figure 3c’). 

**Figure 4:**
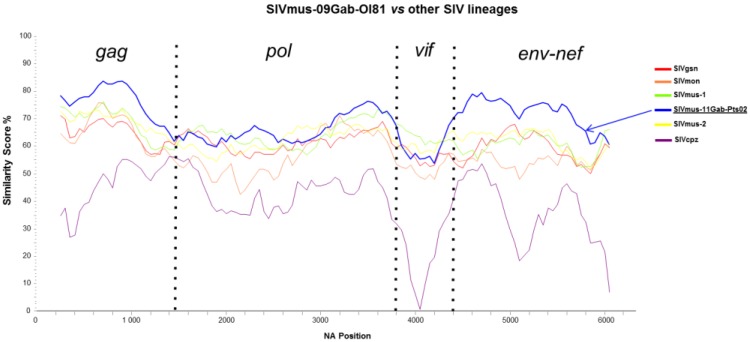
Similarity plots of nucleotide sequences illustrating the extent of genetic diversity between the new SIVmus and other SIV lineages. Similarity plots of *gag*, *pol*, *vif*, *env*, and *nef* non-concatenated nucleic acid sequences showing similarities between SIVmus-09Gab-0I81 and SIVmus-11Gab-Pts02 and other SIVs representative for the different SIV lineages (sliding window of 500 na moved in steps of 50 na). Only SIV lineages of interest are retained in the figure for clarity.

Thus, in order to refine our analyses we performed a similarity plot analysis. Figure 4 shows that, depending on the parts of the genome studied, the new SIVmus strains are most closely related to each other in *gag* and *env* genes whereas in *pol* and *vif* gene they show more divergence and the genomic structure seems more complex as previously suggested by ML phylogenetic analysis in *pol*. Overall, these two new SIVmus strains were closely related to SIVmus-1 & -2, SIVgsn and SIVmon than any other SIV lineages (Figure 4, only SIV lineages of interest were retained in the figure for clarity). These results are strengthened by the identity values obtained between the two new SIVmus strains in Gag, Pol, and Env proteomes (Table 1). In Gag and Env proteomes, the new SIVmus strains shared 89% and 86% of amino acid identity, respectively, whereas in Pol they shared only 76% of amino acid identity. Using nucleic acids, we observed similar results (data not shown).

**Figure 5:**
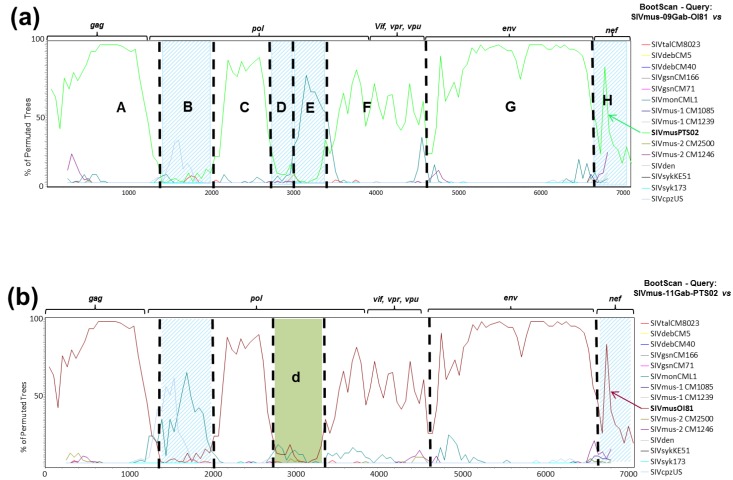
Bootscan analyses of the new SIVmus whole genome sequences *versus* other SIV lineages infecting arboreal *cercopithecus*. Bootscan analyses were performed using the same full-length nucleic acid alignment of SIVs infecting only arboreal *Cercopithecus.* monkeys with the SIMPLOT package version 2.5 [27]. (**a**) showed the bootscan result for SIVmus-09Gab-OI81 *versus* other SIV lineages, (**b**) showed the bootscan result for SIVmus-11Gab-Pts02 *versus* other SIV lineages. Hatched blue rectangles and green rectangle showed the divergent fragments between the two new SIVmus strains (fragments B, D, E, H and d).

In order to further elucidate the complex structure in the *pol* gene, we proceeded to bootscan analyses using solely SIV nucleic acid sequences identified from arboreal *Cercopithecus* monkeys. This enabled us to increase the phylogenetic signal owing to the conservation of around 1000 bp supplementary in the final alignment used for the phylogenetic analyses (~7000 bp *vs.* ~6000 bp when working with a complete SIV alignment). The bootscan analysis results are shown in Figure 5a,b for SIVmus-09Gab-OI81 and SIVmus-11Gab-Pts02, respectively, and showed that both new SIVmus strains were obviously closer in the majority of their genome but seemed to diverge in three different parts of the genome, in *pol* and *nef*.

**Table 1:** Percent amino acid identity between new SIVmus strains (09Gab-OI81 and 11GabPts-02) and SIV strains representative to other SIV lineages in the three major genes Gag, Pol, and Env. Grey rectangles show the percentage of amino acid identity between the two new SIVmus-09Gab-OI81 and -11Gab-Pts02 strains (highlighted in red) and other SIV strains (in bold) from the SIVmus/mon/gsn lineage along the three major genes Gag, Pol, Env.

SIV Strains	Gag		Pol		Env	
	SIVmus	SIVmus	SIVmus	SIVmus	SIVmus	SIVmus
	09GabOI81	11GabPts02	09GabOI81	11GabPts02	09GabOI81	11GabPts02
SIVgsnCM166	**80**	**80**	**74**	**75**	**73**	**74**
SIVgsnCM71	**78**	**77**	**75**	**75**	**74**	**76**
SIVmonCML1	**77**	**75**	**71**	**73**	**69**	**70**
SIVmus-1 CM1085	**81**	**82**	**76**	**74**	**76**	**79**
SIVmus-1 CM1239	**82**	**83**	**75**	**73**	**77**	**78**
SIVmusOI81	**100**	**89**	**100**	**76**	**100**	**86**
SIVmusPTS02	**89**	**100**	**76**	**100**	**86**	**100**
SIVmus-2 CM2500	**84**	**84**	**75**	**72**	**77**	**78**
SIVmus-2 CM1246	**83**	**82**	**72**	**71**	**75**	**76**
SIVtal	75	74	63	65	54	53
SIVdeb	67	66	60	61	45	45
SIVden	65	66	62	63	47	46
SIVrcm	65	64	57	59	42	40
SIVagi	64	63	58	60	40	40
SIVdrl	66	65	58	60	37	37
SIVmnd-2	64	63	57	59	38	39
SIVsyk	68	66	61	61	49	49
SIVcpz*Pts*	57	57	57	59	57	56
SIVlho	52	51	55	55	37	37
SIVsun	53	54	54	54	36	36
SIVmnd-1	56	56	55	55	36	35
SIVwrc*Pbb*	49	50	56	55	35	35
SIVwrc*Pbt*	50	50	54	55	36	36
SIVsmm	61	62	58	58	42	41
SIVagm	63	63	56	58	44	44
SIVcpz*Ptt*	59	59	59	59	52	52
SIVgor	48	49	58	59	53	52
HIV-2	62	63	57	57	42	41
SIVcol	48	48	52	53	35	35
SIVolc	50	50	51	50	36	38

Taking into account these results, we performed more detailed phylogenetic and genetic identity analyses on DNA fragments defined on the basis of bootscan results. Fragments A, B, C, F, G and H were common to both new SIVmus-Gab strains whereas fragments D and E were specific to SIVmus-09Gab-OI81 and fragment d to SIVmus-11Gab-Pts02 (Figure 5). In addition, a substitution saturation test showed that all sequence sets used for the phylogenetic analyses experienced little substitution saturation (*Iss < Iss.c*, data not shown) [33].

**Figure 6:**
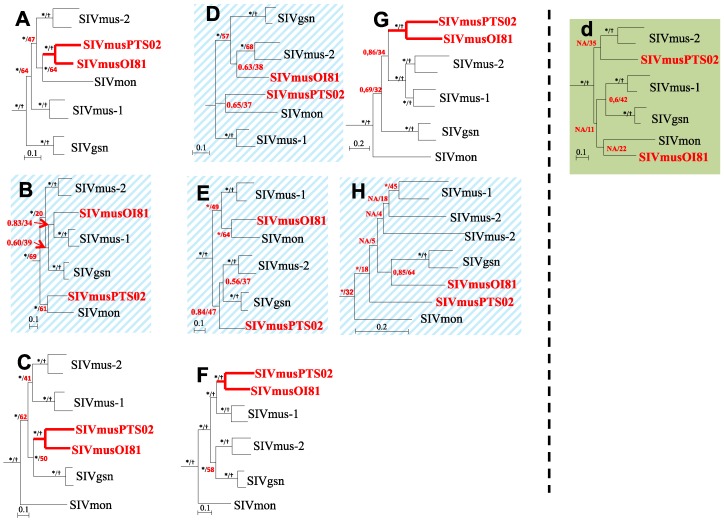
Phylogenetic relationships between SIVmus-09Gab-OI81 and SIVmus-11Gab-Pts02 with other representative SIV lineages infecting arboreal *cercopithecus* along the full-length genome. Phylogenetic analyses were done according to the bootscan analyses (Figure 5). We defined eight fragments (**A** to **H**) for SIVmus-09Gab-OI81 and seven fragments (**A**–**C**, **d**, **F**–**H**) for SIVmus-11Gab-Pts02. Phylogenies were inferred using both Bayesian (run for 1,000,000 generations) and Maximum Likelihood methods implemented in Mr Bayes and PhyML, respectively, under the GTR + Γ_4_ + I model of evolution. Stars and crosses at nodes represent posterior probability (≥90%) and bootstrap (≥70%) values, respectively. Scale bars indicate substitution per site. The new SIVmus strains are highlighted in red.

As shown in Figure 6, except in two different parts of the *pol* gene (B, D/E and d) and in *nef* gene (H), the new SIVmus strains clustered together along their whole genome representing a separate SIVmus lineage (Figure 6). These results were strengthened by the genetic identity values shown in Table 2. We compared the genetic distances of the different SIVs within and between host species. The genetic distance between the new SIVmus strains in the *gag* gene was similar to those observed between the SIVmus-2 strains. In the *pol*, *accessory*, and *env* genes, according to the fragments analyzed, the values varied between 70% and 76% which are the lowest values observed within intra-host species in this SIV lineage (Table 2). Moreover, although the identity values were lower than those for other SIVs within host species, the new SIVmus clade was highly supported (Figure 6).

The phylogenetic tree analysis confirmed that the position of the new SIVmus strain’s fluctuated according to the fragments analyzed. The two new SIVmus strains were closer to SIVmon in the *gag* gene (fragment A), SIVgsn in the middle part of *pol* gene (fragment C), SIVmus-1 in the end of *pol* gene as well as in accessory genes (fragment F). In the envelope (fragment G), the new SIVmus strains were at the root of SIVmus-1 & -2 thus forming all together a specific SIVmus *env* clade (Figure 6). Nonetheless, except for fragment F, the ML topologies were not well sustained in comparison with the Bayesian methods, however the different tree topologies obtained using both Bayesian and ML methods were identical for all fragments except for fragment d and H (data not shown). Moreover the identity values in fragment A and C did not correspond to the tree topologies. Actually, SIVmus-Gab strains seemed closer to SIVmus-1 and -2 than to SIVmon in fragment A whereas in fragment C they were equidistant to SIVmus-2 and SIVgsn (Table 2).

**Table 2:** Percentage of nucleic acid identities within new SIVmus-09Gab-OI81 and -11Gab-Pts02 strains, SIVmus-1, SIVmus-2, SIVgsn lineages and between each of new SIVmus-Gab with SIVs representative to SIVmus/mon/gsn lineage according to the recombinant analysis. Identity values of interest are showed in bold. In fragments A, C, F, and G, identity values show that new SIVmus strains are closely related although in fragment F, SIVmus-09Gab-OI81 is closer to SIVmus-1 than SIVmus-11Gab-Pts02. In the fragment B, SIVmus-Gab strains are equidistant from each other and from all other SIVmus/mon/gsn lineages. In fragment H, both SVmus-Gab are close to SIVmus-1. In fragments D and E, SIVmus-09Gab-OI81 is close to SIVmus-2 and SIVmon, respectively. In fragment d, SIVmus-11Gab-Pts02 seems related to SIVgsn.

	SIV Strains	Fragment									
		**A**	**B**	**C**	D *	E *	d **	**F**	**G**	**H**
**Intra**	SIVgsn (CM166 *vs.* CM71)	89	90	89	91	88	89	90	87	85
**host**	SIVmus-1 (CM1085 *vs.* CM1239)	85	83	81	84	82	82	83	82	79
**species**	SIVmus-2 (CM2500 *vs.* CM1246)	81	78	78	76	81	78	80	79	70
	SIVmus-Gab (GabOI81 *vs.* GabPts02)	**81**	70	**72**	70	73	71	**74**	**76**	73
**SIVmus-09Gab-OI81 *vs.***										
**Inter**	SIVgsn	74	**72.5**	67.5	72.5	72	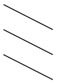	71.5	71	72
**host**	SIVmus-1	76.5	71	62	70.5	73.5		**75.5**	70	**74.5**
**species**	SIVmus-2	75.5	69.5	68.5	**75**	72.5		70.5	70.5	70
	SIVmon	73	66	64	67	**75**		69	68	72
**SIVmus-11Gab-Pts02 *vs.***										
	SIVgsn	74.5	**71**	68	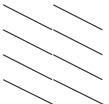		**74.5**	71	68	72
	SIVmus-1	75	70	66			70.5	**72.5**	70	**74.5**
	SIVmus-2	75.5	68.5	70			72	71	70.5	71.5
	SIVmon	72	**71**	64			68	69	68	70

*: Specific fragments used for SIVmus-09Gab-OI81 phylogenetic analyses according to Figure 6; **: Specific fragments used for SIVmus-11Gab-Pts02 phylogenetic analyses according to Figure 6.

The new SIVmus strains did not cluster together in two parts of the *pol* gene and in the *nef* gene. In fragments B and D, SIVmus-09Gab-OI81 clustered with SIVmus-1 and SIVmus-2, respectively, but the nodes were not well sustained by both methods used (M. Bayes and ML) whereas in fragments E and H, SIVmus-09Gab-OI81 seemed closer to SIVmon and SIVgsn, respectively, than other SIVs although the ML bootstrap values were low (64%) (Figure 6). In addition, SIVmus-09Gab-OI81’s genetic distance matched with the phylogenetic position observed in fragments D and E, respectively, with SIVmus-2 and SIVmon. In fragments B and H, the higher genetic identity values observed, 72.5% and 74.5% with SIVgsn and SIVmus-1, respectively, were not correlated with the tree topologies obtained (Table 2, Figure 6).

SIVmus-11Gab-Pts02 was close to SIVmon in fragment B, but here too with a low ML bootstrap value while in fragment d and H the tree topology was unresolved whatever the phylogenetic method used (Figure 6). But in these latter fragments, identity values were 71% with SIVmon and SIVgsn (B), 74.5% with SIVgsn (d) and 74.5% with SIVmus-1 (H) (Table 2), respectively.

## 4. Discussion

We described here the molecular characterization of the full-length genome of two new and divergent SIVmus strains sequenced from mustached monkeys in Gabon. These two viruses were isolated from animals hunted in two different villages, which are 250 km apart. Both villages were situated to the north of Ogooué River falling into the natural habitat of the *C. cephus cephus* subspecies, which expands into Cameroon and Congo.

The natural hosts for SIVmus are animals of the *C. c. cephus* subspecies, which are part of the *cephus* superspecies. Six species and 14 subspecies compose this superspecies. In West Central Africa, one species (*C. cephus*) consisting of four subspecies is present: *C. c. erythrotis* in Cameroon above the Sanaga River and in Bioko Island*, C. c. cephodes* south of the Ogooué River in Gabon, *C. c. ngottoensis* between the Sangha and the Congo-Oubangui Rivers in Congo and *C. c. cephus* inhabiting the largest part of west Central Africa from the south of the Sanaga River to the north of the Ogooué River and following the right bank of the Congo River [18]. Besides SIVmus in *C. c. cephus*, to date SIVs has only been partially characterized in *C.c. erythrotis* on Bioko (SIVreg) [36].

Two different SIVmus variants (SIVmus-1 and SIVmus-2) have already been described in mustached monkeys from South Cameroon, from animals sharing habitats within the same geographic area. In this previous study, we showed that the genetic evolution of the SIVmus/mon/gsn lineage was complex and was driven by inter-lineage recombination of the currently known viruses (*i.e*., SIVmus, SIVmon, and SIVgsn) [7]. We also suggested that (1) SIVmus-2 resulted from cross-species transmission involving SIVmus-1, SIVgsn, and a yet unknown SIV and (2) that the SIVmus-1 and -2 Env sequences could represent a “true” SIVmus lineage [7].

Phylogenetic analyses of these two new SIVmus strains showed that they were distinct to previously reported SIVmus strains but belonged to the SIVmus/mon/gsn lineage. Thus, we confirmed that the *C. cephus cephus* subspecies is the first NHP, known to date, infected by at least three SIVmus variants; SIVmus-1, SIVmus-2, and the new SIVmus strains from Gabon.

Nonetheless, although the new SIVmus strains are quite similar and form a separate cluster in ≈80% of their genome, some differences are observed in the *pol* and *nef* genes confirming a complex evolutionary history within this SIV lineage that involved cross species transmission and recombination events such as previously suggested for SIVmus-1 and -2 [7]. In addition, in the *env* gene, the SIVmus strains from Gabon clustered as outlier of SIVmus-1 and -2. Nonetheless, the node of SIVmus-1, -2 and -musGab was not well sustained and this cluster might be resolved by additional SIV *env* sequences, yet to be discovered, and elucidate the more ancient origin of certain SIVmus clades. Further investigations on SIVs in the *C. cephus cephodes* subspecies and other species potentially infected with the SIVmon/mus/gsn lineage might shed light onto this genomic region as well as investigations for lentiviruses infecting the other NHP species close to mustached monkeys.

In this study, we performed two different phylogenetic approaches: Bayesian and Maximum likelihood analyses. Although the topologies obtained were similar, the reliability of phylogenetic trees was significantly different. The differences observed between posterior probabilities and bootstrap values were not surprising. Indeed, Suzuki *et al.* suggested that the bootstrap probabilities were more suitable for accessing the reliability of phylogenetic trees than posterior probabilities [37]. In addition, certain tree topologies did not match with the genetic identity values obtained. Nonetheless, this is unsurprising facing the low bootstrap values of these phylogenetic trees.

Besides, the lower genetic identity values observed between these two new SIVmus strains when compared to other SIVs within host-species could be explained by a more ancient SIV infection in mustached monkeys from Gabon than in Cameroonian mustached monkeys (Table 2). However, this assertion needs to be further documented. Actually, SIVmus-1 and -2 have been isolated from animals collected in two different sites that are 40 km apart [38]. Also, new studies on SIVmus in Cameroon are needed to evaluate the genetic diversity of this lineage in the country.

All our *C. cephus cephodes* samples were negative for SIV infection. Nonetheless, we only tested 23 samples, thus whether this absence of SIV is representative of the region requires further investigation. Moreover, the overall SIVmus prevalence for *C. cephus cephus* in our study was 6.2%. In Cameroon, 864 mustached monkey samples were tested in eight different sites on samples derived from bush meat displayed on markets and an overall SIV prevalence of only 1.2% is observed [38]. Nevertheless, SIVmus were found in only three sites situated in South-East Cameroon with the prevalences of 0.9%, 1%, and 7.3%. The highest SIVmus prevalence was found in the geographic area where the two SIVmus variants co-circulate (SIVmus-1 & -2).

Of additional interest was the identification of a domain in C1 of the gp120 envelope that has so far been neglected in the analysis of the lentiviral lineage expressing *vpu*. While it was not within the scope of this study to further characterize this domain, it is tempting to speculate that this domain could have functional significance to remain conserved as a surface accessible domain. Whether it is solely of structural nature, such as the interaction with the other gp120 subunits on the envelope surface peak, or whether its function is independent on viral fusion on potentially connected to the presence of the *vpu* gene will require subsequent studies. This domain is likely evolved in a common ancestor of SIVmus/SIVmon/SIVgsn. Remarkably, key features such as the *vpu* and sequence signatures of yet unknown function in the envelope gene remained conserved even after the recombination events that had led to the evolution of SIVcpz/gor and during its subsequent transmissions leading to the different groups of HIV-1. It seems, therefore, reasonable to propose that the investigations of these unique viral features could contribute to a better understanding of HIV-1 and its high virulence.

## 5. Conclusions

These results highlight the need to continue to study and explore SIV infections in monkeys from the same species in different locations to further understand the evolutionary history of these microorganisms. Indeed the *cephus* superspecies represents the largest monkey group inhabiting sub-Saharan Africa [39]. These monkey species are also among the most poached primates in Central Africa, and hunters continue to be extensively exposed to these viruses, which harbor a potential threat for the public health. That precursors to the SIVmus/mon/gsn subset can successfully cross species borders into a hominoid host is suggested by the recombinant origin of SIVcpz/gor.

Finally, in this study we used the classical Sanger sequencing approach and directly sequenced overlapping PCR fragments to obtain the SIVmus full-length genomes. Nonetheless, Lauck *et al*., recently described new and divergent SIV strains that infect *C. guereza* in Uganda using a random hexamer based deep-sequencing approach [40]. This new approach could be more sensitive than amplification with strain specific and/or consensus primers to detect new divergent sequences, but the sensitivity to detect new viruses that are present at low titers remains to be evaluated.

## Supplementary Files

Supplementary File 1 Supplementary Materials (PDF, 756 KB)
