# Supplementary material for: Full-Length Genome Analyses of Two New Simian Immunodeficiency Virus (SIV) Strains from Mustached Monkeys (C. Cephus) in Gabon Illustrate a Complex Evolutionary History among the SIVmus/mon/gsn Lineage
Source: Viruses. 2014 Jul 22;6(7):2880–98. doi: 10.3390/v6072880 (PMC4113797; doi:10.3390/v6072880)
Supplement: Supplementary File 1 — Supplementary Materials (PDF, 756 KB) [file viruses-06-02880-s001.pdf]

Supplementary Materials

Full-Length Genome Analyses of Two New Simian Immunodeficiency Virus (SIV) Strains from Mustached Monkeys (*C. Cephus*) in Gabon Illustrate a Complex Evolutionary History among the SIVmus/mon/gsn Lineage

Florian Liégeois, Fabian Schmidt, Vanina Boué, Christelle Butel, Fatima Mouacha, Paul Ngari, Bertrand Mve Ondo, Eric Leroy, Jonathan L. Heeney, Eric Delaporte, Martine Peeters and François Rouet

Figure S1. Phylogenetic analysis of partial SIV *pol* sequences. Phylogenetic relationships of the newly derived SIVs in partial *pol* sequences to representatives of the other SIV lineages. The newly identified SIVmus strains in this study are highlighted in red. Reference strains are in black except for SIVmus-1 & 2 that are highlighted in orange. The unrooted trees were inferred from 300 bp nucleotides. The analyses were performed using discrete gamma distribution and GTR model. The starting tree was obtained by using phyML. One thousand bootstrap replications were performed to assess confidence in topology. Stars at nodes represent bootstrap (≥70%) values. Scale bar represents the number of nucleotide substitution per site.

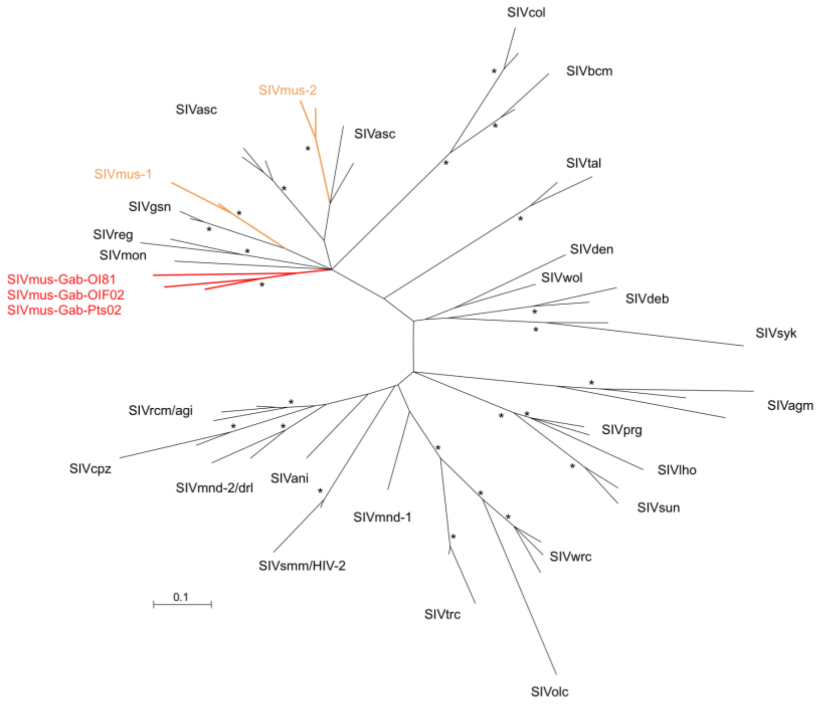

Figure S2. Phylogenetic analysis of partial 12S rRNA sequences from the mustached monkeys collected in Gabon. Reference sequences used were as follow: *C. cephus* (L35191, L35202), *C. diana* (L35193), *C. mona* (L35198), *C. aethiops* (L35187, L35189, L35190), *C. nictitans* (L35199), *C. mitis* (L35197), *C. patas* (L35186), *C. neglectus* (L35182), *C. galeritus* (L35208), *M. sphinx* (L35196), *C. torquatus* (L35204), *Papio species* (L35184, L35206), *L. atterimus* (L35192), *M. talapoin* (L35205), *G. gorilla* (L35209), *P. paniscus* (L35201), *P. troglodytes* (L35183). The analyses were performed using discrete gamma distribution and TN93 model. The starting tree was obtained using PhyML. In red are represented the *C. cephus cephus* subspecies whereas *C. cephus cephodes* are highlighted in blue. Arrows showed animals subspecies misclassified on field. Red rectangles showed animals infected by SIVmus (09Gab-OI81, 11Gab-Pts02, 11Gab-OIF02).

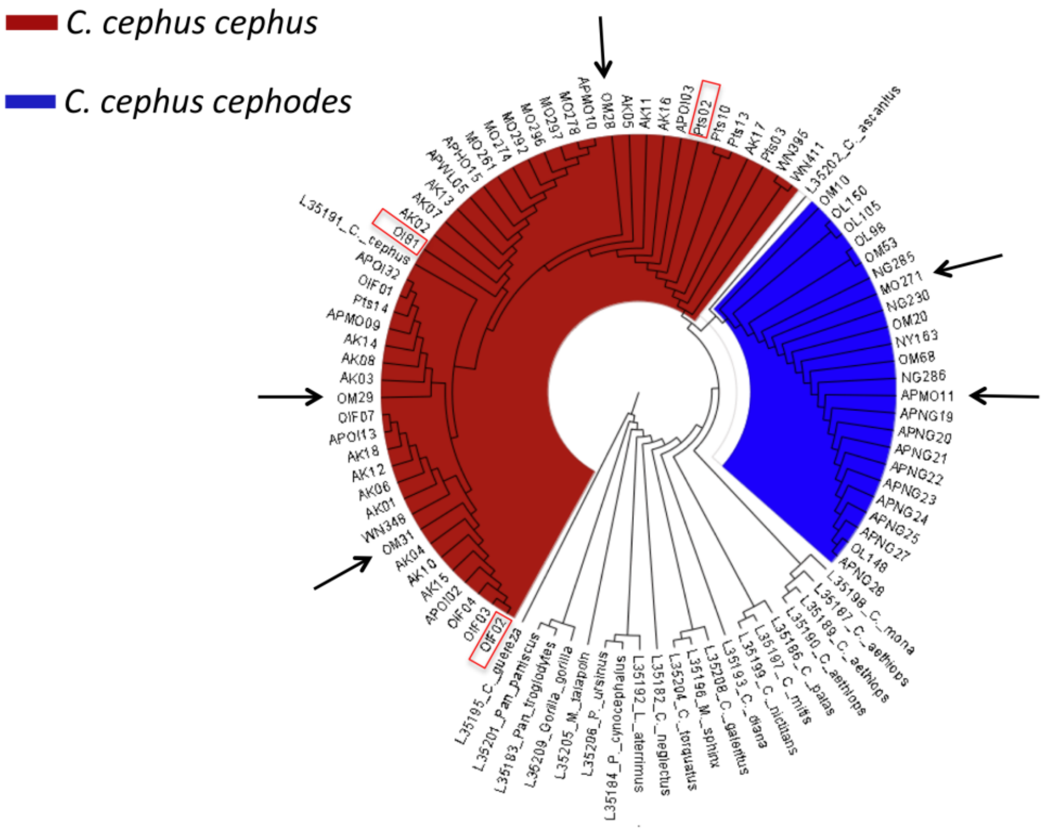

Figure S3. Secondary structure predictions of SIVmus-09Gab-OI81 and -11Gab-Pts02 TAR element. Secondary structure predictions showed that all arboreal *Cercopithecus* SIVs have a duplicated stem-loop structure consisting of a single nucleotide bulge (C or U), a 3-bp stem, and a 6-bp terminal loop with the sequence 5′-CUGGGA-3′. The predicted TAR secondary structures for SIVmus-09Gab-OI81 and 11Gab-Pts02 also exhibited duplicated TAR elements containing a 3-bp stem between the bulge and the terminal loop.

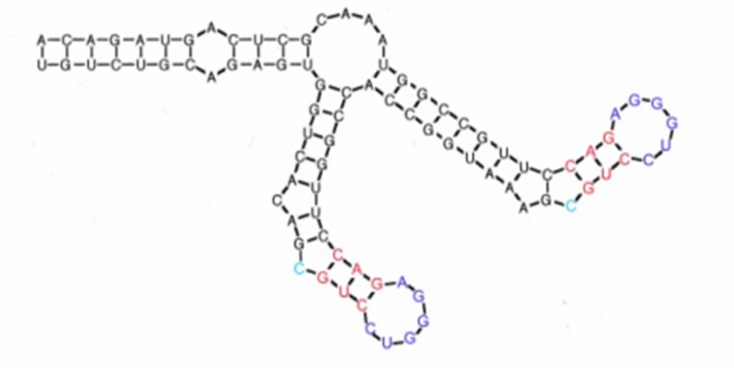

	Loop 1			Loop 2		
	Bulge	bp stem	Loop	Bulge	bp stem	Loop
SIVtal	C	3	CUGGGA	C	3	CUGGGA
SIVdeb	C	3	CUGGGA	U	3	CUGGGA
SIVgsn	C	3	CUGGGA	C	3	CUGGGA
SIVsyk	C	3	CUGGGA	C	3	CUGGGA
SIVmon	C	3	CUGGGA	C	3	CUGGGA
SIVmus	C	3	CUGGGA	C	3	CUGGGA
SIVmus-OI81	C	3	CUGGGA	C	3	CUGGGA
SIVmus-Pts02	C	3	CUGGGA	C	3	CUGGGA

Table S1. Primers used to amplify full-length genomes of SIVmus-09Gab-OI81 and SIVmus-11Gab-Pts02.

Samples	Primers [a]	Size (kb)
	SIVmus-09Gab-OI81	
First round	* Polis4 (5'-CCAGCNCACAAAGGNATAGGAGG-3'); * PolOR (5'-ACBACYGCNCCTTCHCCTTTC-3')	
Second round	* Polis2 (5'TGGCARATRGAYTGYACNCAYNTRGAA-3); * Uni2 (5'-CCCCTATTCCTCCCCTTCTTTTAAAA-3')	0.4
First round	§ OIPolF1 (5'-TAGCAAGTCAATGGCCAGTAAGTC -3'); * SIVenvR (5'-YTBYTGCTGCTGCAMTATCCC -3')	
Second round	* cpz6768 (5'-TTAAYTGTCATGGRGAATTYTTYTAYTG-3'); * SIVenvR	0.5
First round	§ OIPolF1 5'-TAGCAAGTCAATGGCCAGTAAGTC -3'); § OIEnvR3 (5'-GTCTATCTCCACCACTTTGTA-3')	
Second round	§ OIPolF2 (5'-GCAAACAAGCAGCTAAAGACACA-3'); § OIEnvR2 (5'-GCTTTATTTTGCAAGTCATCCA-3')	3.1
First round	§ OIEnvF1 (5'-TGGATGACTTGCAAAATAAAGC-3'); * SIVnef-as (5'-CAGTCCHCCCTTTTCTTT-3')	
Second round	§ OIEnvF2 (5'-TCTAAGTCAATATATCTCCCACCA-3'); * SIVnef-as	1.4
First round	§ OINefF1 (5'-CTCCGCAGAACCCTATCCATC-3'); * SIVgagR (5'-GGNCCTCCCACTCCTTGGCADGC-3')	
Second round	§ OINefF2 (5'-CGGAGGCAACGTCAAATCAG-3'); * SIVgagR	1.5
First round	§ OIGagF1 (5'-ACAGGGACCTAAGGAACCATT-3'); § OIPolR1 (5'-CTTGACTTTGAGGGTTGTATGGTA-3')	
Second round	§ OIGagF2 (5'-CCTAAGGGCAGAACAGACA-3'); § OIPolR2 (5'-GCTGTGTTTCTCCCTGTTTCTCTA-3')	2.8
	SIVmus-11Gab-Pts02	
First round	& PolCMN F1 (5'-TCTYTATCCYTCCCTGTCATCYCTCT-3'); & PolCMNR1 (5'-TCCCCYATTCCTCCCYTYYTTTTA-3')	
Second round	& PolCMN F2 (5'-GACACMGGRGCNGAYGACACCAT-3'); & PolCMNR2 (5'-GCCTGTGCTACTGCTGTTTC-3')	2.4
First round	& EnvCMNF1 (5'-TGTGTVAAAYTGACHCCNATGTGTGT-3'); * SIVnef-as (5'-CAGTCCHCCCTTTTCTTT-3')	
Second round	& EnvCMNF2 (5'-CARATAGGAGCAGGMATGAC-3'); & EnvCMNR1 (5'-TTGGAGTTCTTGGAKCCCAT-3')	2.1
First round	# Pts02-PolF1 (5'-TGGAATCAGGACAAAGCAACT-3'); # Pts02-EnvR1 (5'-CAAGCAAACCAATGTGTCTCTACT-3')	
Second round	# Pts02-PolF2 (5'-TACAATTAACAGAGGCAGCAGAAG-3'); # Pts02-EnvR2 (5'-GGCTTGTCCACCTCATTGTTACT-3')	4.1
First round	# Pts02-EnvF1 (5'-GGAGCTACCAGACCTTGAGAA-3'); # Pts02-PolR1 (5'-CATCTCCTGGGTTATCTGCT-3')	
Second round	# Pts02-EnvF2 (5'-GACGAAGCTGGTGGCAACTCA-3'); # Pts02-Pol R2 (5'-CTGTTACCGGGAAATTTAGTGTGA-3')	3.2

[a] Y = C ou T; W = A ou T; R = A ou G; H = A ou C ou T; B = C ou G ou T; M = A ou C; S = G ou C; K = G ou T; V = G ou A ou C; D = G ou A ou T; N = A ou G ou C ou T, I = inosine; * SIV generic primers, § SIVmus-09Gab-OI81 specific primers; # SIVmus-11Gab-Pts02 specific primers; & SIVmus/mon/gsn/ lineage specific primers.
